# Characterization of *Ascaris* from Ecuador and Zanzibar

**DOI:** 10.1017/S0022149X14000431

**Published:** 2014-06-16

**Authors:** A.M. Sparks, M. Betson, G. Oviedo, C. Sandoval, P.J. Cooper, J.R. Stothard

**Affiliations:** 1 Department of Parasitology, Liverpool School of Tropical Medicine, Pembroke Place, Liverpool, L3 5QA, UK; 2 Department of Production and Population Health, Royal Veterinary College, Hatfield, Herts, AL9 7TA, UK; 3 Laboratorio de Investigaciones FEPIS, Quinindé, Esmeraldas Province, Ecuador; 4 Centro de Investigación en Enfermedades Infecciosas, Pontificia Universidad Católica del Ecuador, Quito, Ecuador; 5 Centre for Infection, St George's University of London, London, SW 17ORE, UK

## Abstract

To shed light on the epidemiology of ascariasis in Ecuador and Zanzibar, 177 adult worms retrieved by chemo-expulsion from either people or pigs were collected, measured and subjected to polymerase chain reaction–restriction fragment length polymorphism (PCR-RFLP) analysis of the ribosomal internal transcribed spacer (ITS) region. Upon double digestion with *Rsa*I and *Hae*III, PCR-RFLP analysis revealed the presence of *A. lumbricoides* in people and *A. suum* in pigs in Ecuador. In contrast, while there are no pigs on Zanzibar, of the 56 worms obtained from people, one was genotyped as *A. suum*. No additional genetic variation was detected upon further PCR-RFLP analysis with several other restriction enzymes. Upon measurement, worm mass and length differed by location and by species, *A. suum* being lighter and longer. While there is no evidence to suggest zoonotic transmission in Ecuador, an enduring historical signature of previous zoonotic transmission remains on Zanzibar.

## Introduction

Ascariasis, caused by infection with the giant roundworm *Ascaris lumbricoides,* is common throughout the developing world and is estimated to affect ~14.6% of the world's population (Brooker & Pullan, 2013). Although the majority of infections are asymptomatic, many millions suffer chronic morbidity (de Silva *et al.*, 1997) with the greatest burden on children aged 5–15 years (Bethony *et al.*, 2006). *Ascaris lumbricoides* is closely related to *Ascaris suum*, the large roundworm of pigs, from which it is morphologically indistinguishable, differing by only 1.9% sequence divergence in the mitochondrial genome (Liu *et al.*, 2012). Laboratory studies have shown that *Ascaris* eggs from infected humans can infect pigs and *vice versa* (Takata, 1951; Galvin, 1968). Consequently, it is uncertain whether these are two true species (Leles *et al.*, 2012) or how transmission might incorporate pigs as a zoonotic reservoir. To distinguish worms and investigate transmission cycles, sequence analysis of mitochondrial DNA markers, particularly *cox*1 and *nad*1 genes, has been used, as well as sizing of microsatellite-containing loci (Betson *et al.*, 2013). A simpler method involving polymerase chain reaction–restriction length polymorphism (PCR-RFLP) analysis of the ribosomal internal transcribed spacer region (ITS) with *Hae*III has revealed a polymorphic restriction site that can broadly differentiate the two species upon distinctive RFLP profiles (Anderson *et al.,* 1995; Nejsum *et al.*, 2005).

To date, molecular epidemiology studies have shown it is highly likely that ascariasis is a zoonosis in North America, Denmark, Brazil, Japan, the United Kingdom and other European countries (Anderson, 1995; Nejsum *et al.*, 2005; Arizono *et al.*, 2010; Bendall *et al.*, 2011; Iñiguez *et al.*, 2012; Cavallero *et al.*, 2013). In addition, zoonotic transmission has been demonstrated in China and Uganda (Zhou *et al.*, 2012; Betson *et al.*, 2014). The global view is far from complete, however, and many countries with high *Ascaris* prevalence have received no attention. Despite a high prevalence of *Ascaris* (Cooper *et al.*, 2003; Moncayo *et al.*, 2008) and widespread pig farming in rural parts of Ecuador, no studies have been published on the molecular epidemiology of *Ascaris* in these areas. In rural areas, where humans live in close contact with pigs and sanitation is poor, there is a clear risk of zoonotic transmission. The objective of the present study was to investigate evidence of cross-transmission of *Ascaris* in Ecuador and Zanzibar by PCR-RFLP analysis of the ITS, using double digestion with *Hae*III and *Rsa*I, further screening for diversity with several other restriction enzymes.

## Materials and methods

**Fig. 1:**
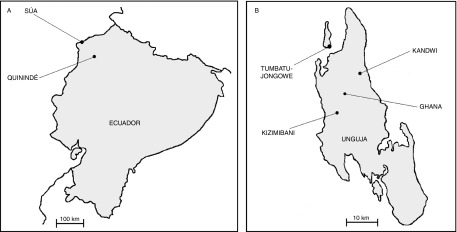
Sources of *Ascaris* used in the study: (A) location of Quinindé where human and pig sampling was carried out and Súa where pig sampling was carried out within Ecuador; and (B) the location of the four villages (Kandwi, Ghana, Kizimbani and Tumbatu-Jongowe) on Unguja, Zanzibar.

A parasitological survey of children and pigs was conducted in May/June of 2013 in Esmeraldas Province, northern coastal Ecuador (fig. 1A) and had been undertaken previously in children in Zanzibar (Stothard *et al.*, 2008) (fig. 1B). Spatial co-ordinates, as taken by a hand-held GPS unit (e-trex, Garmin Ltd, Olathe, Kansas, USA), in decimal degrees, for the villages surveyed were in Ecuador: Quinindé (+0.330618^o^, − 79.464481^o^) and Súa (+0.860764^o^, − 79.875254^o^), and in Zanzibar: Tumbatu-Jongowe ( − 5.855866^o^, − 39.229831^o^), Kandwi ( − 5.943357^o^, − 39.334667^o^), Ghana ( − 6.002032^o^, − 39.260545^o^) and Kizimbani ( − 6.057987^o^, − 39.245278^o^). Infection status with *Ascaris* was determined using direct saline examinations and duplicate standard 41.7 mg Kato–Katz smears. Children with infection intensities of >500 eggs/g of stool were treated with pyrantel pamoate at 10 mg/kg of body weight (Combantrin®, Pfizer, Quito, Ecuador).

**Fig. 2:**
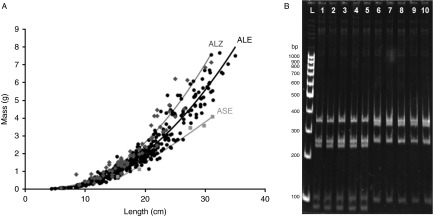
The morphological and molecular characterization of *Ascaris* from Ecuador and Zanzibar. (A) The relationship between worm mass and length of 380 human-derived worms from Ecuador (ALE) of which 172 were subjected to PCR-RFLP, 5 pig-derived worms from Ecuador (ASE) and 68 worms from Zanzibar (ALZ), all with quadratic regression lines. One outlier from the Ecuador data set was removed due to measurement error. (B) PAGE gel image following digestion of ITS PCR products of Ecuadorian *Ascaris* samples with *Hae*III and *Rsa*I. Lanes: L, 100 bp ladder; 1–5, pig-derived worms; 6–10, human-derived worms.

Within Quinindé and Súa, pigs were also examined for infection using duplicate Kato–Katz smears and then treated with piperazine citrate (retailed as S.P. VERMES soluble powder, Qalian, Segre, France) at 0.2 g/kg by delivery in food or water. All expelled stools were collected from treated children and pigs. Worms were removed from stools and washed thoroughly. After blotting dry, the mass and length of each worm was measured twice and data were analysed by regression (fig. 2A). Worms were placed in 70% ethanol for long-term storage for later genotyping.

Genomic DNA was extracted previously from 56 *Ascaris* worms from Zanzibar, originally collected from children in 2006 (Stothard *et al.*, 2008; Betson *et al.*, 2011). The ribosomal ITS was amplified from all worms according to a published PCR protocol (Nejsum *et al.*, 2005). To maximize detection of variation within ITS amplicons, double digestions with *Hae*III and *Rsa*I were performed, and products were separated by non-denaturing polyacrylamide gel electrophoresis (PAGE) with ethidium bromide staining.

## Results and discussion

In Ecuador 381 worms were collected from 22 children, and 5 worms from pigs; a total of 68 worms were collected from 14 individuals in Zanzibar. The mean worm length and mass of human-derived worms was 18.3 cm and 1.97 g in Ecuador and 17.1 cm and 2.07 g in Zanzibar. A clear relationship between worm mass and length was found (*r*_s_= 0.968, *n*= 453, *P*< 0.001; fig. 2A) and it appeared that worms could be differentiated by location: worms from Zanzibar appeared to be heavier than worms from Ecuador. In addition, pig-derived worms appeared to be lighter than human-derived worms from Ecuador. A test for the equality of slopes of log weight on log length (Draper & Smith, 1998) found that the regression lines of each group were parallel (*F*_2,447_= 0.85, *P*= 0.426) but had significantly different intercepts, with pig-derived worms being lighter, and human-derived worms from Zanzibar being heavier, at any given length (*F*_2,447_= 56.18, *P*< 0.001).

*Ascaris* ITS sequences were digested with multiple restriction enzymes, but only *Hae*III was found to have a polymorphic restriction site. A double digestion with restriction enzymes *Hae*III and *Rsa*I was performed because this approach created fragment sizes amenable for PAGE analysis. This double digest produced a four-band human-derived *Ascaris*-like genotype or a five-band pig-derived *Ascaris-*like genotype (fig. 2B). In total, 121 *Ascaris* worms (31.3%) were transported to the UK and genotyped. All 116 worms from children produced the human-derived *Ascaris-*like genotype profile, while all 5 worms from pigs produced the pig-derived *Ascaris-*like genotype profile. In contrast, of the 56 worms from children in Zanzibar that were genotyped using PCR-RFLP, one worm from Kizimbani was found to have a pig-derived *Ascaris*-like genotype.

Genotyping adult worms using an ITS PCR-RFLP assay did not provide any evidence of cross-transmission of *Ascaris* in this sympatric setting in Quinindé, Ecuador, and there appears to be a clear separation of transmission cycles of *A. lumbricoides* in people and *A. suum* in pigs. In Zanzibar, however, only one worm was characterized by PCR-RFLP analysis to be *A. suum*. Previous molecular characterization of these worms by Betson *et al.* (2011), using *cox*1 haplotype analysis, found five worms with pig-derived *Ascaris*-like genotypes, while microsatellite data found no zoonotic worms or hybrids (Betson *et al.*, 2014), illustrating that differing conclusions may have been drawn with other markers (Anderson, 2001). As the population of Zanzibar is Muslim and pigs are no longer present, it is unlikely that pigs are a source of infection in this area. Instead Betson *et al.* (2011) hypothesized that these genotypes are an ancestral genotype which was retained in Zanzibar, or they crossed by introgression from *A. suum* to *A. lumbricoides* worms when pigs were more common in the early twentieth century.

The use of molecular epidemiology is crucial in determining the source of *Ascaris* infections and implementing effective control strategies. In areas where pigs and humans co-exist and where cross-transmission has been found to be a significant source of infections, control programmes must also focus on targeting treatment of pigs. This is important not just in terms of health but because transmission of ascariasis between humans and pigs could allow the spread of drug resistance and virulence alleles into human parasites (Iñiguez *et al.*, 2012). In other parts of the world, such as our study area in Ecuador, where pig holdings are common but where there does not appear to be cross-transmission, control programmes could be simplified to exclude infection control in pigs.

## Acknowledgements

The authors would like to thank the field teams in Ecuador and Zanzibar for assistance.

## Financial support

Financial support was received from the Liverpool School of Tropical Medicine. The work reported here constitutes the MSc research project findings of A.M.S. Sample collection in Ecuador was supported by a Wellcome Trust grant 088862/Z/09/Z.

## Ethical standards

The study protocol was approved by the Ethical Committees of Liverpool School of Tropical Medicine and Pontificia Universidad Catolica del Ecuador.
